# Clinical utility of miniprobe endoscopic ultrasonography for prediction of invasion depth of early gastric cancer: A meta-analysis of diagnostic test from PRISMA guideline: Erratum

**DOI:** 10.1097/MD.0000000000014868

**Published:** 2019-03-08

In the article, “Clinical utility of miniprobe endoscopic ultrasonography for prediction of invasion depth of early gastric cancer: A meta-analysis of diagnostic test from PRISMA guideline”,^[1]^ which appears in Volume 98, Issue 6 of *Medicine*, Mingchi Luo should be the only corresponding author. The corresponding author information should appear as The Second Affiliated Hospital of Tianjin University of Traditional Chinese Medicine, NO. 69, Zengchan Avenue, Hebei District, Tianjin City 300250 China (luomingchimed@foxmail.com).

Mingchi Luo's affiliation should be The Second Affiliated Hospital of Tianjin University of Traditional Chinese Medicine, Tianjin City 300250 China.
